# Translation, cross-cultural adaptation, and psychometric properties of the Finnish version of the Malocclusion Impact Questionnaire (MIQ)

**DOI:** 10.2340/aos.v84.42833

**Published:** 2025-01-30

**Authors:** Lucas Arrais Campos, Terhi Kaikkonen, Kaisa Ylitervo, Leena Ylikontiola, Anna-Sofia Silvola

**Affiliations:** a Faculty of Medicine and Health Technology, Tampere University, Tampere, Finland; b Department of Ear and Oral Diseases, Tampere University Hospital, Tampere, Finland; c Faculty of Health Sciences, Institute of Dentistry, University of Eastern Finland, Kuopio, Finland; d School of Dentistry, São Paulo State University (UNESP), Araraquara, Brazil; e Research Unit of Population Health, University of Oulu, Oulu, Finland; f Wellbeing Services County of Kainuu, Kainuu, Finland; g The Wellbeing Service County of North Ostrobothnia, Pohde, Finland; h Medical Research Center Oulu, Oulu University Hospital and University of Oulu, Oulu, Finland

**Keywords:** Psychometrics, validation studies, malocclusion, questionnaire, oral health-related quality of life

## Abstract

**Objectives:**

This study aimed to translate and adapt the Malocclusion Impact Questionnaire (MIQ) into Finnish; to estimate its psychometric properties when applied to Finnish adolescents; and to estimate the effect of demographic characteristics on the perceived impact of malocclusion.

**Methods:**

The Finnish version of MIQ (MIQ-Fi) was established through translation, back-translation, and a pilot study. Psychometric properties were estimated using factorial validity (confirmatory factor analysis [CFA]), convergent validity (Average Variance Extracted [AVE]), and reliability (α_ordinal_ and ω). Structural Equation Model estimated the effect of demographic characteristics on malocclusion impact.

**Results:**

A total of 268 Finnish adolescents participated in the study (mean age = 13.4 [standard deviation, SD = 1.5] years, 48.5% girls). MIQ-Fi factor model presented an adequate fit to the data after refinements (CFA: comparative fit index [CFI] = 0.96, Tucker-Lewis index [TLI] = 0.95, standardized root mean square residual [SRMR] = 0.08, exclusion of 4 items and 1 correlation between items error). Convergent validity (AVE = 0.61) and reliability (α_ordinal_ and ω ≥ 0.90) were adequate. Gender and self-reported need for orthodontic treatment had moderate effects on malocclusion impact (β_standardized_ = 0.36 and 0.30, respectively, *p* < 0.01), while other demographic characteristics had weak effects (β_standardized_ < |0.18|, *p* < 0.04).

**Conclusion:**

MIQ-Fi demonstrated adequate psychometric properties and can measure malocclusion impact in Finnish adolescents. Demographic characteristics had weak to moderate effect on the malocclusion impact.

## Introduction

Malocclusion refers to an irregularity in teeth position or a misalignment of the dental arches beyond what is considered normal, which may affect function, esthetics, social interaction, and oral health-related quality of life (OHRQoL) [1–3]. Orthodontics is the dental specialty dedicated to treating this clinical condition. Evidence-based orthodontics (EBO) is a patient-centered practice philosophy that replaces the traditional approach by giving equal importance to the patient’s perspective, the orthodontist’s expertise, and scientific evidence [4]. This approach complements traditional expert- and condition-based orthodontics that primarily emphasizes physical and biological measures. A better understanding and inclusion of the patient’s perspective in orthodontic treatment through OHRQoL measures are essential for providing higher-quality dental care [5].

Dental patient-reported outcome (dPRO) is a measure of OHRQoL, encompassing any report regarding the status of a patient’s oral health condition directly from the patient, without the interpretation of others [6, 7]. Due to their subjective nature, dPROs cannot be directly measured [7–10]. Therefore, it is necessary to use standardized instruments, known as dental patient-reported outcome measures (dPROMs), to measure them indirectly [6, 7].

An extensive number of dPROMs can be found in the literature [11]; however, most of them are either generic measures of OHRQoL or condition-specific measures unrelated to malocclusion. Consequently, using dPROMs from different clinical contexts may not adequately capture the impact of malocclusion on a patient’s life [12]. Some dPROMs have been proposed for use in the context of orthodontic treatment. Examples include the Orthognathic Quality of Life Questionnaire [13, 14], developed for orthognathic patients, and the Psychosocial Impact of Dental Aesthetics Questionnaire [15]. Both were originally developed for a target population of young adults (≥18 years). Although young adults are an important group of orthodontic patients, many seeking treatment are often younger.

To address the need for a child-centered, malocclusion-specific dPROM, Malocclusion Impact Questionnaire (MIQ) was proposed [12, 16]. MIQ was developed in English and the content of its items was derived from semi-structured interviews [12]. Adolescents who were interviewed raised three major concerns related to malocclusion: appearance of teeth, effect on social interactions, and oral health and function. Following a pilot study and Item Response Theory (IRT) analysis, the final version of MIQ consists of 17 items composing a single construct that measures the impact of malocclusion on life [16].

Data obtained from MIQ demonstrated adequate validity and reliability when applied to samples of orthodontic patients from the UK [16], New Zealand [17], and Nigeria [18]. The overall MIQ score was able to discriminate between treated and untreated patients [19] and was responsive to outcome changes following orthodontic treatment [20]. MIQ has been translated and culturally adapted into Chinese [21], Spanish [22], Moroccan Arabic [23], Serbian [24], and Saudi Arabian Arabic [25]. These versions also presented satisfactory psychometric properties (validity and reliability) when applied to the target populations.

Those results have demonstrated the current and promising use of MIQ for measuring the perspective of orthodontic patients and contributing to EBO. Therefore, it is pertinent that MIQ be translated and culturally adapted for other languages and countries, and that this impact be measured across different populations, taking into account individual characteristics. The present study aims to translate and culturally adapt MIQ into Finnish (MIQ-Fi); to estimate the psychometric properties of MIQ-Fi when applied to Finnish adolescents; to verify the measurement invariance and discriminative ability of MIQ-Fi between Finnish adolescents who have never received orthodontic treatment and those who have received such treatment; and to estimate the effect of demographic characteristics on the perceived impact of malocclusion. Our specific hypotheses are:

Hypothesis 1: The data obtained with MIQ-Fi are valid and reliable when applied to Finnish adolescents.

Hypothesis 2: MIQ-Fi has measurement invariance between Finnish adolescents who have never received orthodontic treatment and those who have received or are currently receiving orthodontic treatment, and it is able to discriminate between these groups.

Hypothesis 3: Demographic characteristics and self-reported information on orthodontic treatment and satisfaction with occlusal function significantly affect the perceived impact of malocclusion as measured by MIQ-Fi.

## Methods

### Study design and sampling

This was a cross-sectional study with a non-probabilistic sample of Finnish adolescents. Participants were recruited from two schools located in two municipalities in Finland (Raahe and Suomussalmi). The inclusion criteria for the study were individuals aged between 11 and 17 years. Adolescents who did not respond to one or more questions related to demographic information or items comprising the factor model of the MIQ were excluded from the analyses.

The minimum sample size was calculated based on the proposal of Hair et al. [10], which set a minimum of 10 respondents per manifest variable to be estimated in the model. As the structural model has 23 manifest variables to be tested (17 items + 6 independent variables), the minimum sample size was 230 participants. Considering a loss rate of 15%, the minimum sample size to be recruited was 271 adolescents.

### Procedures and ethical aspects

The data collection was conducted during the school day between February and April 2022. Participants self-completed the questionnaires using the pencil-and-paper method. For ethical approval, an initial consultation was conducted with the Regional Medical Research Ethics Committee of the Wellbeing Services County of North Ostrobothnia. The Ethics Committee informed that, according to the Medical Research Act of the country where the study was conducted, approval from the regional medical research ethics committee is not required for survey-based studies that do not interfere with the integrity of the research subjects.

Approval for data collection was obtained from the Data Protection Officer at the University of Oulu, in accordance with the European Union’s General Data Protection Regulation. The parents or legal guardians of the adolescents were informed in advance about the study before data collection, during which they had the chance to decline the adolescent’s participation. Subsequently, only the adolescents who agreed and consented participated in the study.

### Study variables

The following demographic information was collected for sample characterization: age, gender, and self-reported orthodontic treatment history or ongoing orthodontic treatment. In addition, self-assessed orthodontic treatment need and the level of satisfaction with occlusion function were measured using a number rating scale from 0 to 10. As the participants are adolescents, socioeconomic status was not assessed.

MIQ was used to measure the perceived impact of malocclusion. Also, the Finnish version of Psychosocial Impact of Dental Aesthetics Questionnaire (PIDAQ-Fi) [26] was used to verify the validity of MIQ. These psychometric scales are described below.

### Measurement scales

MIQ [12, 16] has 17 items constituting a one-factor model (items 3–19) and 2 global questions (items 1 and 2). Responses to the 17 items of the unifactorial model are based on a 3-point severity scale (0: don’t or doesn’t, 1: a bit, and 2: very or a lot). The original proposal for obtaining a total score is by summing the individual item scores, ranging from 0 to 34. The response to the 2 global questions is on a 5-point severity scale (0: not at all, 1: a little bit, 2: a bit more, 3: a lot, and 4: very much) and their scores are presented separately and are not considered in the total score.

PIDAQ-Fi [26] was used to assess the validity of MIQ based on relations to another variable. This scale consists of 24 items distributed into 4 factors (dental self-confidence, social impact, psychological impact, and esthetic concern). The response scale is a 5-point Likert-type scale ranging from 0 to 4 (0: I do not agree, 1: I agree a little, 2: I somewhat agree, 3: I agree a lot, 4: I totally agree).

### Translation and cross-cultural adaptation of MIQ into Finnish

Three independent translators translated the English version of the MIQ into Finnish. One translator was professional and two were specialist dentists and native speakers of Finnish. The translations were compared by two researchers who have prepared a preliminary Finnish version. The version was back-translated into English by another independent professional translator. Two researchers compared the original, preliminary, and back-translated versions. Minor changes were made to the preliminary Finnish version [27].

A pilot study was conducted using the preliminary version (MIQ-Fi). The Incomprehension Index (II) was estimated. This index aims to verify any difficulties by the participants in understanding the content of items. If the values of II for the items are lower than 15%, the version is considered adequate and the final one [28].

### Psychometric properties of the Finnish version of MIQ

The validity and reliability evidence of the data obtained with MIQ-Fi was verified following the Standards for Educational and Psychological Testing proposal [29]. Validity was considered based on response process, internal structure, relation to another variable, and consequence validity.

#### Validity based on the response process

The validity based on the response process was assessed using IRT analyses considering the Rasch Model for Polytomous Data. The analysis was conducted using the Winsteps software (Winsteps^®^ Version 5.6.0, Portland, Oregon, USA) [30]. Reliability indicators for individuals and items were evaluated, and values above 0.70 were considered adequate. Information-weighted mean square (infit) and unweighted mean square (outfit) indices assessed deviations in participants’ performance [30, 31]. Infit verifies unexpected response patterns from individuals who exhibit a latent trait (θ) equivalent to the item’s difficulty level. Outfit, on the other hand, verifies unexpected response patterns from those with a θ different from the item’s difficulty level. Infit and outfit estimates were evaluated using mean square (MNSQ) and standardized z (ZSTD) indicators. For MNSQ, values between 0.5 and 1.5 logits indicate that the data fits the model [32]. For ZSTD, values above |2| indicate that the estimate does not fit the data appropriately.

Differential Item Functioning analysis (DIF) was conducted between participants who had never received orthodontic treatment and those who had received or were undergoing orthodontic treatment to verify if this treatment could affect the item response pattern. For this purpose, the Wald test [33] and the likelihood ratio test [34] were used. The difference between the item difficulty estimates between the groups was used as the effect size of the DIF. A significance level of 5% was adopted and the false discovery rate (FDR) adjusted *p*-value was considered for controlling type I errors in multiple comparisons.

#### Validity based on internal structure

The validity based on internal structure was assessed by factorial and convergent validity. Initially, the psychometric sensitivity of the items was examined using descriptive statistics of the responses to the items composing the factor model of MIQ-Fi. Absolute values of skewness and kurtosis lower than 3 and 7, respectively, were indicative of no severe violation of normal distribution [8], confirming the psychometric sensitivity of the item [9].

The factorial validity was estimated through Confirmatory Factor Analysis [CFA] using the robust estimation method Weighted Least Squares Mean and Variance Adjusted (WLSMV). The fit indices considered were the comparative fit index (CFI), the Tucker-Lewis index (TLI), the root mean square error of approximation (RMSEA), and the standardized root mean square residual (SRMR). The local fit was also estimated considering the factor loading (λ). Adequate model fit to the data was considered when CFI and TLI > 0.90, RMSEA < 0.10, SRMR < 0.08, and λ ≥ 0.50 [9, 35]. If necessary, the modification indices, estimated by Lagrange Multipliers (LM), were inspected to verify the existence of a correlation between item errors (LM > 11) [9].

Convergent validity was evaluated based on the Average Variance Extracted (AVE). Values of AVE ≥ 0.50 were considered adequate [36]. The suggestion of the unidimensionality of MIQ-Fi was evaluated considering the Unidimensional Congruence (UniCo), Explained Common Variance (ECV), and Mean of Item Residual Absolute Loadings (MIREAL) indices. Values of UniCo > 0.95, ECV > 0.85, and MIREAL < 0.30 suggested that the items can be treated as components of a single dimension [37].

The reliability of the data was assessed by considering internal consistency and reproducibility (test-retest). Internal consistency was evaluated using the ordinal alpha (α) and omega (ω) coefficients. Values of α and ω equal to or greater than 0.70 were considered indicative of adequate reliability [9]. For the reproducibility measure, 30 participants responded to the MIQ for a second time after 4–6 weeks. Then, the score at each time point was calculated by computing the mean of the responses to the items that were part of the factor model fitted to the data (after CFA). Test-retest reliability was assessed using the intraclass correlation coefficient (ICC) for absolute agreement, employing the 2-way mixed-effects model [38]. ICC values above 0.75 indicate good reliability [38].

After identifying the factor model that fits the sample data, the total MIQ-Fi score for each participant was calculated based on the mean of the responses to the items included in the final refined factor model. This approach ensures the score reflects the refined factor structure of the scale [9]. Descriptive statistics for the total score (ranging from 0 to 2) were calculated.

The measurement invariance of MIQ factor model was verified between participants who had never received orthodontic treatment and those who had received or were undergoing orthodontic treatment. This analysis allows for certifying the maintenance of the factor model to measure the self-perceived impact of malocclusion in these subsamples. First, the CFA was performed for each subsample. If configural invariance was observed between the subsamples, a multigroup analysis using the CFI difference (∆CFI) was performed to verify the measurement invariance. The WLSMV estimation method was used considering ∆CFI between the configural (M0) and metric (M1) models (∆CFI_M1-M0_), metric (M1) and scalar (M2) models (∆CFI_M2-M1_), and between scalar (M2) and strict (M3) models (∆CFI_M3-M2_). A decrease in CFI (∆CFI) above 0.01 was considered indicative of the absence of measurement invariance [9, 39].

If measurement invariance was observed, the scores of MIQ-Fi were compared between these groups to verify whether MIQ-Fi discriminates between teenagers who had never received orthodontic treatment and those who had received or were undergoing orthodontic treatment. The score calculation was the mean of the responses given to the items that compose the factor model fitted to the data. The skewness and kurtosis estimated the distribution of the scores, and absolute values below 3 and 7, respectively, were indicative of non-severe violation of normal distribution [8]. The scores showed a distribution close to the normal distribution (skewness ≤ |1.5| and kurtosis ≤ |2.4|). The scores had homoscedasticity between the subsamples (Levene’s test: *F* = 1.00, *p* = 0.318); therefore, the comparisons were performed using a *t*-test with equal variances. The significance level adopted was 5%.

Psychometric sensitivity, reproducibility, and score comparisons were conducted using the IBM SPSS Statistics 28 (IBM Corp., Armonk, NY, USA) software. Confirmatory factor analysis, AVE, internal consistency, and measurement invariance were conducted using *lavaan* package [40] in R program (R Core Team, 2022). The analysis of the suggestion of unidimensionality was conducted using the program Factor 11.05 for Windows [41].

#### Validity based on relations to another variable

The fit of the factor model of PIDAQ-Fi was initially verified. Item 6 of this scale did not meet the psychometric sensitivity assumption (skewness = 4.0, kurtosis = 17.5), and therefore, it was not included in the factor model. PIDAQ-Fi presented a satisfactory fit to the data (CFI = 0.94; TLI = 0.93; RMSEA = 0.04; SRMR = 0.05; α ≥ 0.72; ω ≥ 0.71).

The validity of MIQ-Fi based on another measure was assessed through correlational analysis (*r*) between MIQ-Fi factor and the factors of PIDAQ-Fi. A strong and negative correlation (negative convergent validity) is expected between MIQ-Fi and the dental self-confidence factor of PIDAQ-Fi. Conversely, a strong and positive correlation (positive convergent validity) is expected between MIQ-Fi factor and the other factors of PIDAQ-Fi (social impact, psychological impact, and esthetic concern). The analyses were conducted using *lavaan* package [40] in R program (R Core Team, 2022).

#### Consequence validity

Considering the results obtained in the previous analyses, the ethical consequences and quality of the measures obtained from MIQ-Fi in Finnish adolescents were evaluated.

### Structural equation model

A structural equation model was elaborated to address the last objective of the study. The independent variables included gender (0 = male, 1 = female), age (in years), self-report of undergoing orthodontic treatment (0 = no, 1 = yes), self-report of having received past orthodontic treatment (0 = no, 1 = yes), self-assessed orthodontic treatment need (visual analog scale – VAS), and the level of satisfaction with occlusal function (VAS). MIQ-Fi factor was considered the dependent variable. The fit of the structural model was assessed using goodness-of-fit indices mentioned in the section *Validity based on internal structure* . The significance of the path estimates (β) was evaluated using the *z*-test (α = 5%). Values of β_standardized_ less than 0.2 are considered weak, between 0.2 and 0.5 are considered moderate, and greater than 0.5 are considered strong path estimates. This analysis was conducted using *lavaan* [40] and *semTools* [42] packages in R program (R Core Team, 2022).

## Results

### Translation and cultural adaptation of MIQ into Finnish

The pilot study consisted of 30 adolescents with a mean age of 13.6 (standard deviation [SD] = 1.8) years, of whom 66.7% were girls. Among them, 40.0% reported currently undergoing orthodontic treatment at the time of participation, while 36.7% reported having received some orthodontic treatment in the past. All items of MIQ-Fi presented an II of 0.0%. Therefore, the intermediate version obtained after translation, cultural adaptation, and back-translation was considered the final version of MIQ-Fi.

### Study participants

A total of 285 adolescents participated in the study. Among these participants, 17 were excluded because they did not answer all MIQ-Fi items or did not provide some of the demographic information. Therefore, 268 participants were included in the analyses. The mean age was 13.4 (SD = 1.5) years, with 48.5% being girls. Eighty-four adolescents (31.3%) reported undergoing orthodontic treatment at the time of completion of the questionnaires, while 80 (29.9%) reported having undergone orthodontic treatment in the past.

### Psychometric properties of the Finnish version of MIQ

#### Validity based on the response process

**Figure 1:**
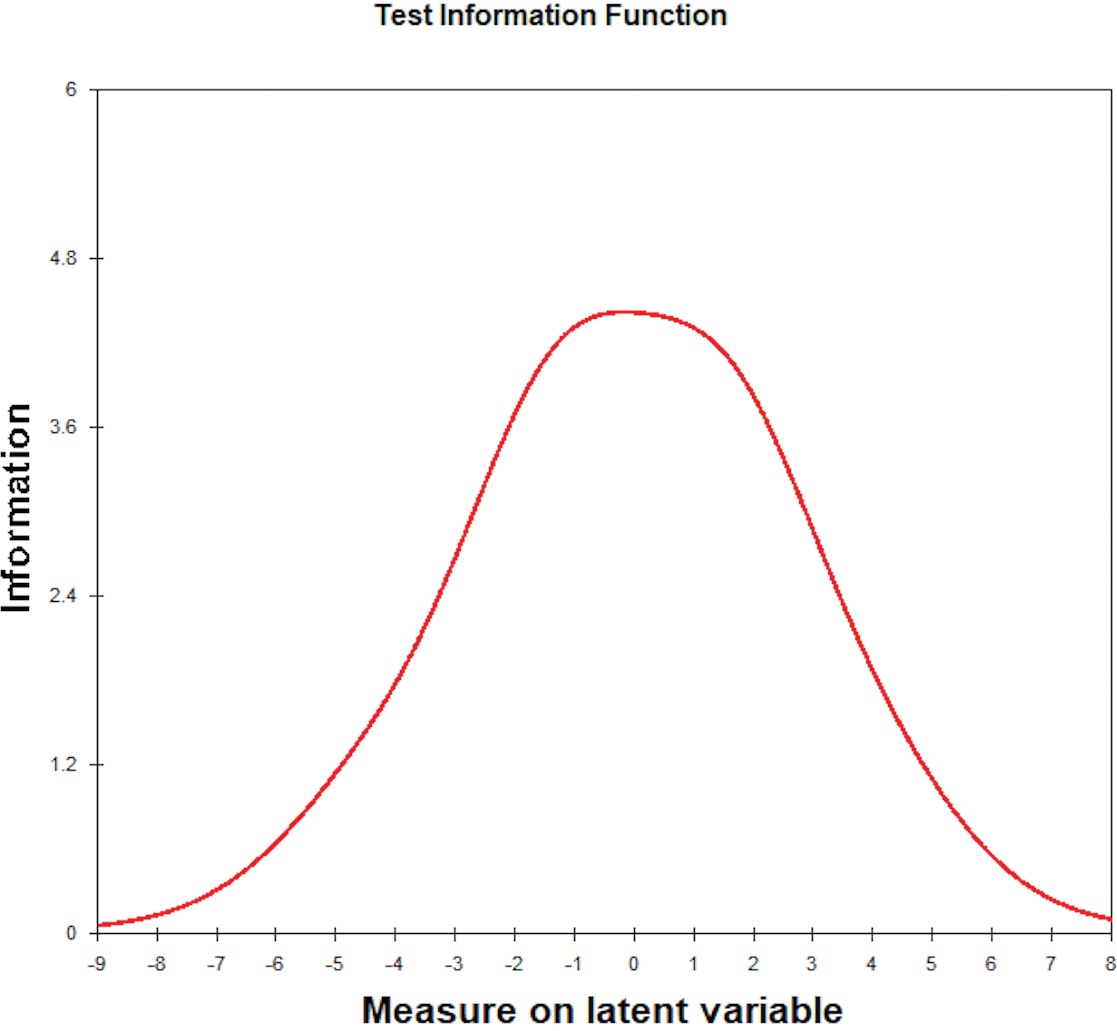
Person-item map of the Finnish version of Malocclusion Impact Questionnaire. Note – Black circles: item difficulty level. White circles: item thresholds.

**Figure 2:**
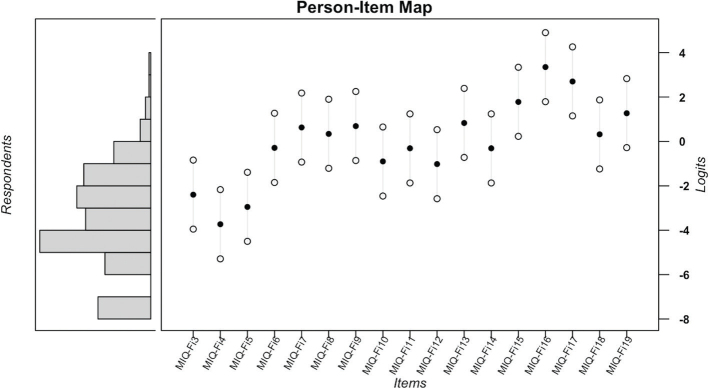
Test information function of the Finnish version of Malocclusion Impact Questionnaire.

The analyses showed adequate reliability indices for the items (reliability = 0.99; separation index = 8.58) and individuals (reliability = 0.79; separation index = 1.92). The person-item map (Figure 1) indicates that, on average, the sample exhibited a latent trait lower than the item difficulty (mean θ = −3.45, mean item difficulty = 0). Most items are located within ±1 logit, enhancing the precision of θ estimates for participants allocated within this range of the latent continuum. This is supported by the Test Information Curve presented in Figure 2.

In Figure 1, it is also observed that items 15 (item difficulty = 1.78), 16 (item difficulty = 3.35), and 17 (item difficulty = 2.70) were the most difficult ones to be endorsed by participants, whereas items 3 (item difficulty = −2.40), 4 (item difficulty = −3.73), and 5 (item difficulty = −2.95) were the easiest. Table 1 presents infit and outfit estimates. It is observed that only item 19 exhibited estimate values above the expected cutoff points. This suggests that this item may degrade the measurement system of the MIQ for the study sample.

**Table 1:** Item fit statistics (information-weighted mean square [infit] and unweighted mean square [outfit]) of the Finnish version of Malocclusion Impact Questionnaire (MIQ-Fi).

Item	Infit		Outfit	
	MNSQ	ZSTD	MNSQ	ZSTD
MIQ-Fi3	1.05	0.57	0.98	−0.13
MIQ-Fi4	0.98	−0.19	1.03	0.31
MIQ-Fi5	0.95	−0.63	0.92	−0.74
MIQ-Fi6	1.18	1.69	1.05	0.30
MIQ-Fi7	0.84	−1.29	0.67	−1.29
MIQ-Fi8	0.80	−1.79	0.63	−1.79
MIQ-Fi9	1.20	1.55	1.06	0.30
MIQ-Fi10	0.82	−2.00	0.81	−1.32
MIQ-Fi11	0.94	−0.57	0.88	−0.57
MIQ-Fi12	1.17	1.71	1.04	0.34
MIQ-Fi13	0.98	−0.07	0.85	−0.35
MIQ-Fi14	0.71	−3.08	0.62	−2.22
MIQ-Fi15	1.09	0.55	0.82	−0.19
MIQ-Fi16	0.95	−0.06	1.62	0.99
MIQ-Fi17	0.96	−0.07	0.80	−0.13
MIQ-Fi18	1.15	1.25	0.82	−0.65
MIQ-Fi19	1.67	3.82	3.77	3.98

ZSTD: standardized z; MNSQ: mean square.

Appendix 1 presents the results of the DIF analysis between participants who have never received orthodontic treatment (*n* = 104) and those who have received or are undergoing orthodontic treatment (*n* = 164). Items 16, 17, and 19 were automatically excluded from the analysis by the software because their response patterns were considered inappropriate for the subsamples, either due to low variability (Appendix 2) or inadequate fit. The Wald test was not statistically significant for any item threshold (Appendix 1), suggesting that the items are responded to similarly, regardless of whether the participants have received orthodontic treatment or not. The likelihood ratio test was also not statistically significant (*χ*² [14] = 16.36, *p* = 0.292).

#### Validity based on internal structure

The descriptive statistics of the responses to MIQ items are presented in Table 2. Responses to items 15, 16, and 17 presented high skewness and kurtosis absolute values, indicating a severe violation of normal distribution, not meeting one of the assumptions of the CFA analysis. This aligns with the findings from the IRT, demonstrating that these items were the most difficult to endorse, with little variability in responses. The covariance matrix of MIQ factor model, including all items, was not positive (Table 3). Faced with these results, the decision was made to exclude these items from the factor model.

**Table 2:** Descriptive statistics of participant responses to items in the Finnish version of Malocclusion Impact Questionnaire.

Item	Mean	Standard deviation	Median	Minimum	Maximum	Skewness	Kurtosis
MIQ-Fi1	0.88	0.92	1	0	4	1.05	0.96
MIQ-Fi2	0.50	0.78	0	0	4	1.68	2.59
MIQ-Fi3	0.74	0.69	1	0	2	0.40	−0.88
MIQ-Fi4	1.06	0.67	1	0	2	−0.06	−0.77
MIQ-Fi5	0.87	0.72	1	0	2	0.20	−1.05
MIQ-Fi6	0.32	0.52	0	0	2	1.34	0.83
MIQ-Fi7	0.19	0.44	0	0	2	2.19	4.18
MIQ-Fi8	0.23	0.48	0	0	2	2.00	3.28
MIQ-Fi9	0.19	0.44	0	0	2	2.28	4.61
MIQ-Fi10	0.42	0.58	0	0	2	1.01	0.03
MIQ-Fi11	0.32	0.56	0	0	2	1.57	1.49
MIQ-Fi12	0.44	0.66	0	0	2	1.19	0.20
MIQ-Fi13	0.17	0.41	0	0	2	2.24	4.34
MIQ-Fi14	0.32	0.51	0	0	2	1.20	0.34
MIQ-Fi15	0.09	0.33	0	0	2	3.73	14.41
MIQ-Fi16	0.03	0.19	0	0	2	7.08	55.38
MIQ-Fi17	0.05	0.23	0	0	2	5.10	28.08
MIQ-Fi18	0.23	0.52	0	0	2	2.20	3.95
MIQ-Fi19	0.13	0.35	0	0	2	2.47	5.05

MIQ-Fi: Finnish version of Malocclusion Impact Questionnaire. Items MIQ-Fi1 and MIQ-FI2 are not included in the factor model and have a 5-point severity scale (0 to 4). The remaining items are based on a 3-point severity scale ranging from 0 to 2.

**Table 3:** Fit of the factor model of the Finnish version of Malocclusion Impact Questionnaire applied to adolescents.

MIQ-Fi model	Excluded items	CFA					AVE	*r* items 3 and 5	α	ω
		CFI	TLI	RMSEA	SRMR	λ				
**Complete (all items)^a^**	-	-	-	-	-	-	-	-	-	-
**Refinement 1^b^**										
Total sample	15, 16, and 17	0.95	0.94	0.09	0.09	0.29–0.88	0.58	-	0.94	0.91
Non-Ortho sample	15, 16, and 17	0.96	0.96	0.09	0.11	0.35–0.92	0.60	-	0.94	0.91
Ortho sample	15, 16, and 17	0.96	0.95	0.08	0.10	0.29–0.88	0.58	-	0.94	0.91
**Refinement 2^c^**										
Total sample	15, 16, 17, and 19	0.94	0.93	0.11	0.09	0.62–0.88	0.62	-	0.95	0.91
Non-Ortho sample	15, 16, 17, and 19	0.96	0.96	0.10	0.10	0.52–0.93	0.64	-	0.95	0.91
Ortho sample	15, 16, 17, and 19	0.96	0.95	0.09	0.10	0.69–0.88	0.62	-	0.95	0.92
**Refinement 3**										
Total sample	15, 16, 17, and 19	0.96	0.95	0.09	0.08	0.63–0.88	0.61	0.57	0.95	0.90
Non-Ortho sample	15, 16, 17, and 19	0.98	0.97	0.07	0.09	0.53–0.94	0.62	0.62	0.95	0.89
Ortho sample	15, 16, 17, and 19	0.96	0.95	0.09	0.09	0.69–0.89	0.62	0.54	0.95	0.90

CFA: confirmatory factor analysis; CFI: comparative fit index; TLI: Tucker-Lewis index; RMSEA: root mean square error of approximation; SRMR: standardized root mean square residual; λ: factor loading; AVE: average variance extracted; α: Ordinal alpha coefficient; ω: omega coefficient.

a The covariance matrix was not positive; thus, refinement was carried out by excluding items that did not meet the psychometric sensitivity criterion (items 15, 16, and 17).

b Item 19 had the lowest factor loading.

c The highest value of the modification index (Lagrange Multipliers [LM]) was observed between errors of items 3 and 5 (Total sample: LM = 40.6; Non-Ortho sample: LM = 30.6; Ortho sample: LM = 14.9).

The fit of MIQ-Fi factor model is presented in Table 3. Although the model without items 15, 16, and 17 presented some good fit indices, item 19 had a low factor loading (λ = 0.29). This is consistent with the IRT findings, where this item showed high infit and outfit values. Therefore, as this item may be hindering measurement, it was also excluded from the model. After this refinement, the factor model showed a limited fit to the data, with RMSEA and SRMR values above cutoff points (Table 3). Inspecting modification indices, the highest value was observed between items 3 and 5 (LM = 40.6). Therefore, a correlation between the errors of these items was added. The RMSEA and SRMR indices showed a slight improvement, rendering this new model considered to have an acceptable fit to the data.

Furthermore, this latest factor model presented adequate convergent validity (Table 3). The data reliability was also satisfactory, both for internal consistency (Table 3) and test-retest reliability (ICC = 0.98, 95% Confidence Interval [CI]: 0.83–0.96). Values of UniCo = 0.97, ECV > 0.87, and MIREAL = 0.26 were observed, confirming the unidimensionality of the MIQ.

The mean total score of the MIQ-Fi, considering the items included in the latest refined factor model (i.e. excluding items 15, 16, 17, and 19), was 0.42 (95% CI = 0.38–0.47, standard deviation = 0.37, median = 0.31, first quartile = 0.15, third quartile = 0.67, minimum = 0.00, maximum = 1.85, skewness = 1.19, kurtosis = 1.43). This score is located on the lower end of the scale, indicating that Finnish teenagers had a low perceived impact of their teeth on their lives. However, a total of 5 outliers were observed, that is, participants scoring higher than 1.45, showing that some teenagers were more affected by their teeth compared to the rest of the sample.

Subsequently, CFA was conducted on the subsamples of adolescents who had never received orthodontic treatment (*n* = 104) and those who had received or were undergoing orthodontic treatment (*n* = 164). It was also observed that items 15, 16, and 17 did not show adequate psychometric sensitivity (high skewness and kurtosis values) for both subsamples (Appendix 2). Therefore, these items were excluded from the analysis. Table 3 also presents the fit of MIQ-Fi factor model for these subsamples. The refinements for an adequate fit of the factor model to the subset were the same as those performed for the total sample: additional exclusion of item 19 due to a low factor loading (λ = 0.29–0.35) and the addition of correlation for the error of items 3 and 5 (LM = 14.9–30.6).

The refined factor model presented invariance measurement between the subsamples separated according to orthodontic treatment (∆CFI_M1-M0_ = −0.005, ∆CFI_M2-M1_ = −0.001, and ∆CFI_M3-M2_ = −0.001). The mean score (excluding responses to items 15, 16, 17, and 19 and ranging from 0 to 2) for adolescents who had never received orthodontic treatment was 0.42 (standard deviation = 0.38, 95% CI = 0.35–0.50) and for those who had received or were undergoing orthodontic treatment was 0.43 (standard deviation = 0.38, 95% CI = 0.37–0.48). No difference was observed between the scores (*t*-test: *t* = 0.52, *p* = 0.959), indicating that MIQ-Fi does not discriminate Finnish teenagers according to whether they have received orthodontic treatment.

#### Validity based on relations to another variable

A strong correlation was observed between MIQ-Fi and PIDAQ-Fi factors, confirming negative convergent validity (MIQ-Fi vs. *Dental Self-Confidence*: *r* = −0.81, *p* < 0.01) and positive convergent validity (MIQ-Fi vs. *Social Impact*: *r* = 0.71, *p* < 0.01; MIQ-Fi vs. *Psychological Impact*: *r* = 0.79, *p* < 0.01; MIQ-Fi vs. *Esthetic Concern*: *r* = 0.73, *p* < 0.01).

#### Consequence validity

The evidence of validity and reliability points to negative consequences in clinical decision-making and drawing conclusions when using MIQ-Fi without a careful analysis of its psychometric properties, especially when applied to populations and contexts different from those originally proposed. In the present study, the exclusion of 4 items was necessary. Keeping them in the factor model and score calculation could not produce an estimate that closely approximates reality.

### Structural equation model

The fit of the structural model was considered satisfactory, although the SRMR value was on the cutoff threshold (CFI = 0.96, TLI = 0.98, RMSEA = 0.05, SRMR = 0.09). All independent variables presented statistically significant pathways (p < 0.04), see Table 4. Women, older adolescents, those who had never received any orthodontic treatment, those who self-reported a higher need for orthodontic treatment, and those with lower satisfaction with dental function presented a higher level of impact of malocclusion in their lives (see Table 4). However, it is noteworthy that only gender and self-reported need for orthodontic treatment showed a moderate path estimate (β_standardized_ = 0.36 and 0.30, respectively), while the remaining variables had a weak estimate (β_standardized_ < |0.18|). The model showed an explained variance for malocclusion impact of 30.6%.

**Table 4:** Path estimates of the structural model elaborated to assess the effect of individual characteristics on malocclusion impact assessed using the Finnish version of Malocclusion Impact Questionnaire in Finnish adolescents.

Independent variable	B	β	SE	*p*
Gender	0.86	0.36	0.17	<0.001
Age	0.13	0.17	0.06	0.021
With ongoing orthodontic treatment	−0.44	−0.17	0.21	0.041
With previous orthodontic treatment	−0.46	−0.18	0.21	0.028
Self-reported need for orthodontic treatment	0.11	0.30	0.03	<0.001
Satisfaction level with occlusion function	−0.10	−0.16	0.04	0.019

B: non-standardized path estimate; β: standardized path estimate; SE: standard error.

Explained variance for malocclusion impact: 30.6%. Gender: 0 = male, 1 = female; age: continuous variable in years; ongoing orthodontic treatment: 0 = no, 1 = yes; previous orthodontic treatment: 0 = no, 1 = yes; self-assessed need for orthodontic treatment: continuous variable (visual analog scale), satisfaction level with occlusion function: continuous variable (visual analog scale).

## Discussion

This study was proposed in light of the importance of evaluating the perceived impact of malocclusion for EBO. It translated and culturally adapted MIQ into Finnish (MIQ-Fi) following international translation standards [27]. Hypothesis 1 of the study was accepted, as the data obtained with MIQ-Fi were found to be valid and reliable when applied to Finnish adolescents. Additionally, Hypothesis 2 was partially supported, as MIQ-Fi factor model presented measurement invariance between the subsamples separated according to whether they had received orthodontic treatment or not. However, part of Hypothesis 2 was rejected, as MIQ-Fi was not able to discriminate between these groups. Demographic characteristics, as well as self-reported information on orthodontic treatment and satisfaction with occlusal function, had an effect on the impact of malocclusion on life, thus confirming Hypothesis 3.

To obtain adequate indicators of validity and reliability for the data collected using MIQ-Fi, it was necessary to exclude four items and add the correlation between the errors of two items. The first step involved the exclusion of three items related to social interaction (items 15 – ‘being bullied’, 16 – ‘making friends’, and 17 – ‘fitting in with friends’). This decision was made due to the non-convergence of the covariance matrix in the CFA when all items were present, the severe violation of the normal distribution of responses, and the high difficulty level of these items (IRT analysis). These findings may be related to characteristics of the sample that may affect responses to these items. The Finnish adult population has demonstrated high levels of loneliness and social isolation [43, 44], which may impact social interactions and diminish the importance of dental appearance in these interactions [26, 45]. Although generational differences may exist, children tend to replicate social attitudes and behaviors learned from primary socialization or observed in adults [46, 47], such as those attitudes towards orofacial appearance.

The sample for the present study also included schoolchildren, some of whom may or may not have varying degrees of malocclusion. Additionally, the sample is from two small towns (Raahe: ~24,500 inhabitants; Suomussalmi: ~7,600 inhabitants) located far from Finland’s more densely populated areas. Suomussalmi, for example, has a public health system that offers orthodontic treatment for all children, which is not the reality for most municipalities in Finland. These sample characteristics may result in lower latent traits (i.e. perceived impact of malocclusion) than observed solely in patients just before treatment or in the whole Finnish adolescent population. Consequently, some items may be more difficult to endorse, which may affect how a dPROM captures the latent trait [48]. The decision to conduct the present study using this sample was based on the studies by Bourzgui et al. [23] and Kolawole et al. [18], who observed adequate psychometric indicators of MIQ for non-clinical school samples. Moreover, we intended to provide and test a dPROM that could also be useful for public health indices in Finland and applicable beyond the clinical context.

The exclusion of item 19 (‘biting some foods’) was another refinement due to high values in Item Fit statistics in the IRT analysis and low factor loading in the CFA. This non-fit may be explained by a response bias or a theoretical differential of item content. Item 19 refers to the impact of malocclusion on the orofacial function aspect of OHRQoL, while the other MIQ-Fi items refer to the appearance of teeth (orofacial appearance aspect of OHRQoL). The last refinement in the factor model involved the inclusion of a correlation between the errors of items 3 (‘happy’) and 5 (‘confident’). The justification for allowing correlated errors, in addition to the LM value observed in the CFA, lies in the theoretical proximity between confidence and happiness for schoolchildren, with self-confidence being a potential predictor of self-reported happiness [49, 50].

The refined unifactorial model of MIQ-Fi adequately fitted to the data obtained from Finnish adolescents. When examining sub-samples of adolescents who had received orthodontic treatment and those who had not, it was observed that MIQ-Fi items were responded to similarly and that the refined factor model was invariant. In addition, MIQ-Fi did not discriminate between these samples. This result differs from what was expected, which was the discriminative capacity of the scale. This might be true if the administration setting for adolescents with orthodontic treatment experience were a dental clinic. The setting where a questionnaire is administered can influence responses [51]. Responding to the MIQ-Fi in a clinical setting may make teenagers more aware of their appearance and potentially affect their responses. In the present study, however, all participants completed the questionnaire in the school setting, which, combined with the sociocultural characteristics of the Finnish sample, might explain the lack of discriminative capacity.

It is worth noting that, to the best of our knowledge, this was the first study estimating the psychometric properties of MIQ in which refinements in the factor model were necessary. This difference may be related to the specific characteristics of the Finnish sample, as discussed above, as well as to the methodological differences. In contrast to previous studies [17, 21–25], the present study adhered to the Standards for Educational and Psychological Testing [29], which include various validity analyses that provide a comprehensive view of the items’ functionality in capturing the latent construct reflected in the responses. Thus, the decision to refine the model is based on a set of evidence and theory rather than a single isolated result. Moreover, the refinements do not imply a reduced version of the scale for future use; rather, they represent an analytical step to ensure the validity and reliability of data in the study sample and population [9].

The need for the exclusion of items for the factor model fit sparks debate about how the scale score is calculated and interpreted [48]. According to the original proposal [12, 16], the score for MIQ is calculated by summing the responses to the items, ranging from 0 to 34. By excluding items, the score range decreases; in the case of the results found in the present study, it ranges from 0 to 26. This alters the metric of responses, making comparability and interpretability different between studies using the MIQ. An alternative would be to use the arithmetic mean of the responses to the items in the fitted factor model. Although this approach maintains the metric (from 0 to 2) regardless of the number of excluded items, it assumes that all items reflect the latent construct equally, which is unrealistic [28]. There is still a bias in considering all items of a dPROM as equally important for score computation, where the level of the latent trait can be over or underestimated [28, 52]. Thus, the best recommendation would be to use an overall weighted score obtained from the factor weights of the items derived from either CFA or IRT analysis [9, 28]. However, these factor weights are specific to each study sample and target population, making the score calculation method of a dPROM more difficult and complex for both research and, especially, clinical contexts. In addition to presenting factor weights, future studies should propose approaches to facilitate the practical application of score calculations that are more accurate for specific populations.

As a result of the structural equation model, it was observed that older adolescents and girls experienced a greater impact of malocclusion in their lives. This can be explained by the appearance of teeth as one of the major concerns related to malocclusion and the pattern of body image development [53]. Adolescence represents a phase during which individuals seek their individuality and transition to independence, including forming bonds and developing a sense of belonging to a peer group [54]. Physical appearance plays an important role in this phase. As individuals progress through adolescence, they tend to have a more negative body image until they reach emerging adulthood, where their body image stabilizes around a more positive perception [53]. Malocclusion can impair appearance, leading adolescents to have physical features different from their peers and what is socially established as normal. This can result in higher face dissatisfaction and appearance pressure, which may be reflected in the greater impact of malocclusion on their lives.

For girls, in addition to the developmental phase, a greater esthetic pressure than boys can be observed and explained by the theory of objectification of the female body [55]. This theory proposes that society views and judges women as objects based on their physical appearance. From childhood, girls internalize this social construct, resulting in self-objectification [55, 56]. They begin to see and evaluate themselves primarily from an external perspective, excessively concerned about their appearance [55, 56]. Thus, minor deviations from what is socially considered normal occlusion can affect girls’ body image, leading to dissatisfaction and a greater impact on their lives.

However, these explanations are speculative due to controversy in the literature regarding the effect of demographic characteristics on the impact of malocclusion in adolescents. While Tsichlaki et al. [19] reported a gender effect on this impact, other studies have not found any significant effect of either gender [18, 20, 25] or age [18–20, 25]. The variations in results among the studies may arise from differences in sampling and analytical techniques employed, as well as a genuine sociocultural difference among the study populations. It is worth noting that MIQ is a recently developed scale [12, 16] and is the only child-centered malocclusion-specific dPROM available in the literature. Few studies have been conducted to assess the impact of demographic characteristics on MIQ scores [18–20, 25], which restricts the discussion of this effect. Therefore, standardized cross-national studies that incorporate sociocultural variables should be conducted to gather evidence on this topic across different countries.

Self-reported need for orthodontic treatment and satisfaction level with occlusion function significantly contributed to the impact of malocclusion. It is worth noting that both variables deal with subjective measures and can be considered as part or consequence of the construct evaluated by MIQ. The observed results in this study may be attributed to the similarity of subjectivity and collinearity between these variables and the dependent variable. Our purpose in incorporating these variables into the structural model was to evaluate the explanatory power of straightforward questions, which can be easily administered in dental clinics, in elucidating the impact of malocclusion on adolescents’ lives. Despite observing a moderate path estimate, the complete structural model (including all study variables) explained approximately only 30% of the variability in malocclusion impact. This indicates that merely asking patients about their perceived need for orthodontic treatment and their satisfaction level with occlusal function is insufficient to measure and explain this impact. Therefore, the use of standardized methods such as MIQ or other dPROMS is still necessary for accurately assessing the perceived impact of malocclusion by patients.

The cross-sectional study design can be mentioned as a limitation of the study, which does not allow for causal inference between the variables in the structural model. Convenience sampling can also be pointed out as a limitation. However, this is a commonly used and accepted strategy for validation studies of dPROMs [16, 17, 21, 22, 24, 26]. Furthermore, as discussed above, the sample selection from two cities may limit the generalizability of the results to the general population of Finnish adolescents. Even so, from a theoretical perspective, the refinements in the factor model align with the attitudes and perceptions of Finnish adults regarding OHRQoL observed in previous studies [26, 45]. Therefore, it can be speculated that the results of the present study captured and reflected the peculiarities and common attitudes of the Finnish population. Nevertheless, it is crucial to conduct confirmatory analyses in future studies applying the Finnish version of MIQ to samples from different locations in Finland.

Additionally, the absence of detailed clinical evaluations and objective malocclusion indices of the participants can be considered a limitation. Including clinical variables could provide relevant data to establish associations with the self-perceived impact of malocclusion and offer additional psychometric properties estimates of MIQ-Fi, such as responsiveness. Thus, future investigations using MIQ-Fi in clinical settings are of great relevance to enhance its applicability in both research and clinical practice.

Despite its limitations, this study presented and attested to the psychometric properties of MIQ-Fi. It is expected that MIQ-Fi can serve as a clinical tool for orthodontists in Finland to obtain relevant information for evidence-based practice and patient-centered treatment. Furthermore, this scale is expected to be valuable in public health settings, enabling the incorporation of patients’ perspectives alongside traditional clinical assessments and expert opinions. This approach may ensure that public funds are allocated to prioritized treatments based not only on clinical criteria but also on patient input. Finally, MIQ-Fi is a malocclusion-specific PRO available in the pool of standardized scales for measuring OHRQoL and can be employed in research settings. This is important for evaluating therapies in clinical studies and conducting multicenter, cross-national research aimed at a deeper understanding of the sociocultural effects on the psychosocial impact of malocclusion.

## Conclusion

The data obtained using MIQ-Fi was valid and reliable after refining the factorial model. Therefore, this scale may be useful for measuring the impact of malocclusion in Finnish adolescents within a clinical, research, and public health context. Demographic characteristics had a weak to moderate effect on the impact of malocclusion in Finnish adolescents.

## Supplementary Material

Translation, cross-cultural adaptation, and psychometric properties of the Finnish version of the Malocclusion Impact Questionnaire (MIQ)

Translation, cross-cultural adaptation, and psychometric properties of the Finnish version of the Malocclusion Impact Questionnaire (MIQ)
