# Supplementary material for: Translation, cross-cultural adaptation, and psychometric properties of the Finnish version of the Malocclusion Impact Questionnaire (MIQ)
Source: Acta Odontol Scand. 2025 Jan 30;84:42833. doi: 10.2340/aos.v84.42833 (PMC11808814; doi:10.2340/aos.v84.42833)
Supplement: Translation, cross-cultural adaptation, and psychometric properties of the Finnish version of the Malocclusion Impact Questionnaire (MIQ) [file AOS-84-42833-s1.pdf]

Supplementary material has been published as submitted. It has not been copyedited or typeset by Acta Odontologica Scandinavica.

Appendix 1. Item difficulty estimates of the Finnish version of the Malocclusion Impact Questionnaire (MIQ-Fi) for subsamples of Finnish adolescents who have never received orthodontic treatment (Group 1, n=104) and those who have received or are currently undergoing orthodontic treatment (Group 2, n=164), and estimates from the Differential Item Functioning (DIF) analysis.

				DIF		
Item*	Threshold	G1	G2	Contrast	Wald	p-value
MIQ-Fi3	t1	1.74	1.85	-0.11	-0.40	0.84
	t2	0.54	0.73	-0.19	-0.36	0.84
MIQ-Fi4	t1	2.94	3.10	-0.16	-0.53	0.84
	t2	2.94	3.23	-0.29	-0.57	0.84
MIQ-Fi5	t1	2.00	2.53	-0.53	-1.95	0.43
	t2	1.05	2.09	-1.04	-2.04	0.43
MIQ-Fi6	t1	-0.29	-0.15	-0.14	-0.46	0.84
	t2	-3.53	-3.27	-0.26	-0.38	0.84
MIQ-Fi7	t1	-1.14	-1.06	-0.08	-0.22	0.86
	t2	-5.23	-5.09	-0.14	-0.17	0.86
MIQ-Fi8	t1	-1.06	-0.68	-0.38	-1.09	0.72
	t2	-5.06	-4.33	-0.73	-0.93	0.72
MIQ-Fi9	t1	-0.90	-1.33	0.44	1.22	0.72
	t2	-4.74	-5.63	0.89	1.11	0.72
MIQ-Fi10	t1	0.49	0.31	0.18	0.64	0.84
	t2	-1.97	-2.35	0.38	0.61	0.84
MIQ-Fi11	t1	-0.29	-0.11	-0.18	-0.59	0.84
	t2	-3.53	-3.19	-0.34	-0.49	0.84
MIQ-Fi12	t1	0.54	0.48	0.06	0.22	0.86
	t2	-1.88	-2.02	0.14	0.23	0.86
MIQ-Fi13	t1	-1.06	-1.45	0.40	1.07	0.72
	t2	-5.06	-5.87	0.81	0.98	0.72
MIQ-Fi14	t1	-0.01	-0.31	0.30	1.00	0.72
	t2	-2.96	-3.58	0.62	0.91	0.72
MIQ-Fi15	t1	-1.74	-2.62	0.88	1.87	0.43
	t2	-6.42	-8.20	1.78	1.73	0.44
MIQ-Fi18	t1	-1.23	-0.55	-0.68	-1.93	0.43
	t2	-5.40	-4.07	-1.34	-1.68	0.44

*Items 16, 17, and 19 were automatically excluded from the analysis by the software because their response patterns were considered inappropriate for the subsamples
