# Supplementary material for: Translation, cross-cultural adaptation, and psychometric properties of the Finnish version of the Malocclusion Impact Questionnaire (MIQ)
Source: Acta Odontol Scand. 2025 Jan 30;84:42833. doi: 10.2340/aos.v84.42833 (PMC11808814; doi:10.2340/aos.v84.42833)
Supplement: Translation, cross-cultural adaptation, and psychometric properties of the Finnish version of the Malocclusion Impact Questionnaire (MIQ) [file AOS-84-42833-s2.pdf]

Supplementary material has been published as submitted. It has not been copyedited or typeset by Acta Odontologica Scandinavica.

Appendix 2. Descriptive statistics of the responses to items of the Finnish version of the Malocclusion Impact Questionnaire (MIQ-Fi) by the participants according to subsamples of Finnish teenagers who had received prior orthodontic treatment or not.

Never received orthodontic treatment (n=104) / Have received or are currently undergoing orthodontic treatment (n=164)							
Item	Mean	Median	Standard Deviation	Minimum	Maximum	Skewness	Kurtosis
MIQ-Fi1	0.84/0.90	1/1	0.86/0.95	0/0	4/4	1.16/0.98	2.02/0.51
MIQ-Fi2	0.44/0.53	0/0	0.76/0.80	0/0	3/4	1.75/1.65	2.44/2.75
MIQ-Fi3	0.75/0.74	1/1	0.65/0.72	0/0	2/2	0.30/0.45	-0.69/-0.97
MIQ-Fi4	1.07/1.06	1/1	0.70/0.65	0/0	2/2	-0.09/-0.06	-0.93/-0.63
MIQ-Fi5	0.82/0.91	1/1	0.69/0.74	0/0	2/2	0.26/0.15	-0.89/-1.14
MIQ-Fi6	0.31/0.32	0/0	0.52/0.52	0/0	2/2	1.45/1.30	1.22/0.72
MIQ-Fi7	0.19/0.20	0/0	0.44/0.44	0/0	2/2	2.25/2.16	4.51/4.05
MIQ-Fi8	0.20/0.25	0/0	0.47/0.49	0/0	2/2	2.32/1.82	4.83/2.53
MIQ-Fi9	0.22/0.17	0/0	0.44/0.44	0/0	2/2	1.71/2.67	1.85/6.73
MIQ-Fi10	0.45/0.41	0/0	0.57/0.59	0/0	2/2	0.83/1.12	-0.31/0.27
MIQ-Fi11	0.31/0.33	0/0	0.58/0.56	0/0	2/2	1.73/1.45	2.01/1.18
MIQ-Fi12	0.46/0.44	0/0	0.70/0.64	0/0	2/2	1.20/1.17	0.09/0.24
MIQ-Fi13	0.20/0.15	0/0	0.45/0.38	0/0	2/2	2.14/2.27	3.99/4.26
MIQ-Fi14	0.36/0.30	0/0	0.54/0.49	0/0	2/2	1.17/1.19	0.39/0.18
MIQ-Fi15	0.14/0.07	0/0	0.37/0.30	0/0	2/2	2.74/4.79	7.25/24.22
MIQ-Fi16	0.05/0.02	0/0	0.26/0.14	0/0	2/1	5.90/7.21	37.48/50.60
MIQ-Fi17	0.05/0.05	0/0	0.26/0.22	0/0	2/1	5.90/4.20	37.48/15.82
MIQ-Fi18	0.18/0.27	0/0	0.48/0.54	0/0	2/2	2.67/1.96	6.50/2.91
MIQ-Fi19	0.13/0.14	0/0	0.33/0.36	0/0	1/2	2.30/2.54	3.36/5.76

Note: Slash punctuation marks were inserted between the estimates to separate the values obtained for each subsample.
